# Red blood cell count in cerebrospinal fluid was correlated with inflammatory markers on the seventh postoperative day and all associated with the outcome of aneurysmal subarachnoid hemorrhage patients

**DOI:** 10.3389/fmed.2024.1408126

**Published:** 2024-05-27

**Authors:** Jie Min, Yongfeng Zhao, Chenxi Lv, Hang Hu

**Affiliations:** ^1^Neurointensive Care Unit, The First Affiliated Hospital of Yangtze University, Jingzhou, China; ^2^Department of Hematology, The First Affiliated Hospital of Yangtze University, Jingzhou, China

**Keywords:** aneurysmal subarachnoid hemorrhage, systemic immune inflammation index, system inflammation response index, neutrophil-lymphocyte ratio, cerebrospinal fluid

## Abstract

**Background:**

Exploring factors associated with the outcome of patients with aneurysmal subarachnoid hemorrhage (aSAH) has become a hot focus in research. We sought to investigate the associations of inflammatory markers and blood cell count in cerebrospinal fluid with the outcome of aSAH patients.

**Methods:**

We carried a retrospective study including 200 patients with aSAH and surgeries. The associations of neutrophil, lymphocyte, neutrophil-lymphocyte ratio (NLR), platelet-lymphocyte ratio (PLR), systemic immune inflammation index (SII), system inflammation response index (SIRI), and blood cell count in cerebrospinal fluid on the 1st and 7th postoperative days with the outcome of aSAH patients were investigated by univariate analysis and multivariate logistic regression model.

**Results:**

According to the modified Rankin scale (mRS) score, there were 147 patients with good outcome and 53 patients with poor outcome. The neutrophil, NLR, SIRI, and SII levels on the seventh postoperative day in patients with poor outcome were all significantly higher than patients with good outcome, *P* < 0.05. The multivariate logistic regression model including inflammatory markers and blood cell counts in cerebrospinal fluid on the 1st postoperative day confirmed that red blood cell count in cerebrospinal fluid (≥177 × 10^9^/L; OR: 7.227, 95% CI: 1.160–45.050, *P* = 0.034) was possibly associated with poor outcome of aSAH patients, surgical duration (≥169 min), Fisher grade (III–IV), hypertension, and infections were also possibly associated with the poor outcome. The model including inflammatory markers and blood cell counts in cerebrospinal fluid on the 7th postoperative day confirmed that red blood cell count in cerebrospinal fluid (≥54 × 10^9^/L; OR: 39.787, 95% CI: 6.799–232.836, *P* < 0.001) and neutrophil-lymphocyte ratio (≥8.16; OR: 6.362, 95% CI: 1.424–28.428, *P* = 0.015) were all possibly associated with poor outcome of aSAH patients. The NLR (*r* = 0.297, *P* = 0.007) and SIRI (*r* = 0.325, *P* = 0.003) levels were all correlated with the count of red blood cells in cerebrospinal fluid.

**Discussion:**

Higher neutrophil-lymphocyte ratio and higher red blood cell count in cerebrospinal fluid were all possibly associated with poor outcome of patients with aneurysmal subarachnoid hemorrhage. However, we need a larger sample study.

## Introduction

Stroke is one of the leading causes of disability and death worldwide, of which aneurysmal subarachnoid hemorrhage is more common. A community study on deaths and recurrent vascular events among 500,000 Chinese adults who experienced their first stroke showed that subarachnoid hemorrhage accounted for 2% of strokes (1). Among them, the mortality and disability rate of aneurysmal subarachnoid hemorrhage were relatively high. The mortality of 4,506 patients with higher Hunt-Hess grade aneurysmal subarachnoid hemorrhage (aSAH) from 85 clinical studies was 34% (2). Exploring the prognostic factors of patients with aSAH is a hot research direction. But there is limited research on the association of neutrophil to lymphocyte ratio (NLR), and red blood cell count in cerebrospinal fluid with the outcome of patients. A few studies confirmed the association of NLR with the outcome of aSAH patients. Patients with higher NLR at admission had poorer clinical outcome (3, 4). On the other hand, cerebral vasospasm induced by hemoglobin released from dissolved red blood cells after hemorrhage possibly played an important role in delayed ischemia and poor outcome (5). Our study aimed to explore the associations of neutrophil to lymphocyte ratio and red blood cell count in cerebrospinal fluid with clinical outcome in patients with aSAH, and further investigate whether there was an association between red blood cell count in cerebrospinal fluid and NLR level.

## Methods

### Study design and participants

**Figure 1:**
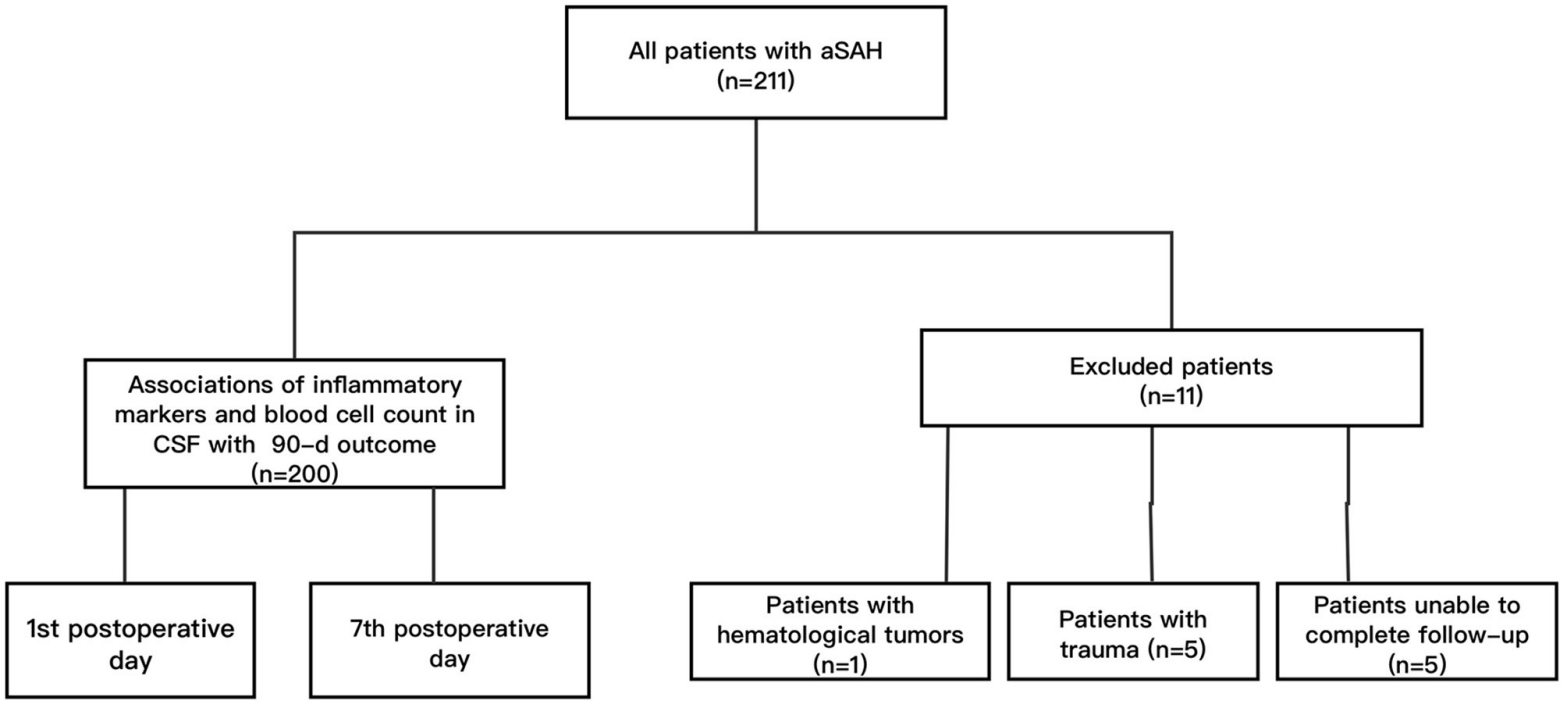
The flow chart of the study. Among 200 included patients, there were 147 patients with good outcome and 53 patients with poor outcome according to the mRS score.

There were 200 surgical patients with aSAH from the Neurointensive Care Unit in the First Affiliated Hospital of Yangtze University from 2020 to 2023 included in this retrospective study. Inclusion criteria: (1) age ≥30 years old; (2) with subarachnoid hemorrhage caused by ruptured aneurysms confirmed by Computed Tomographic Angiography (CTA) and/or Digital Subtraction Angiography (DSA); (3) received endovascular embolization or craniotomy within 48 h of admission. Exclusion criteria: (1) had subarachnoid hemorrhage caused by trauma or unexplained; (2) suffered from serious systemic diseases before admission, such as liver and kidney dysfunction, hematological tumors; (3) with modified Rankin scale (mRS) score before admission more than 2; (4) unable to complete follow-up. This research has been reviewed and approved by the Ethics Committee of hospital. The clinical outcome was determined by the mRS results from 90-day follow-up. The patients with mRS score of 0–2 were determined to have a good outcome, and patients with mRS score of 3–6 were determined to have a poor outcome (6). The flowchart was showed in Figure 1.

### Data collection

Baseline characteristics included age, gender, and comorbidities (hypertension and diabetes), surgical method, surgical duration, Hunt Hess grade, and Fisher grade were also recorded. Blood cells counts including neutrophils, lymphocytes and platelets on the first and seventh postoperative days, and counts of cells in cerebrospinal fluid were extracted from the hospital information system. The neutrophil-lymphocyte ratio (NLR) and platelet-lymphocyte ratio (PLR) were calculated. Systemic immune inflammation index (SII) and system inflammation response index (SIRI) were also calculated. SII = platelet count × NLR. SIRI = monocyte count × NLR.

### Statistical analysis

SPSS 23.0 and GraphPad Prism 6.0 were used for data analysis and graphics. The receiver operating characteristic curve (ROC) was used for the cut-off value analysis. The qualitative variables were compared by Pearson chi-square test, Continuity correction or Fisher exact tests. The quantitative variables were compared by independent-sample *t*-test, corrected *t*-test, or Mann-Whitney *U*-test. The multivariate logistic regression model was constructed for factors associated with the 90-d outcome of patients with aneurysmal subarachnoid hemorrhage. Covariates that had a *P*-value < 0.05 in the univariate analysis were added to the multivariate logistic regression model. All *P*-values were 2-sided and the statistical significance was set at *P* < 0.05.

## Results

Among the 200 patients included, there were 78 males and 122 females. The average age was 60 years old. There were 35 patients with GCS score < 11, 88 patients with intraventricular hemorrhage, 67 patients with Hunt-Hess grade III-V, and 72 patients with Fisher grade III-IV. There were 165 patients with embolization surgeries and 35 patients with craniotomy surgeries. The median surgical duration was 170 min. There were 107 patients with hypertension and 26 patients with diabetes (Table 1).

**Table 1:** Baseline characteristics data in included patients with aneurysmal subarachnoid hemorrhage.

**Characteristics**	**Total (*n* = 200)**	**Good outcome (*n* = 147)**	**Poor outcome (*n* = 53)**	***P-*value**
**Gender [*****n*** **(%)]**				
Male	78 (39.0%)	52 (35.4%)	26 (49.1%)	0.080
Female	122 (61.0%)	95 (64.6%)	27 (50.9%)	
Age (years)	60 ± 10	59 ± 10	62 ± 9	0.019
Surgical duration (minutes)	170 ± 80	160 ± 70	180 ± 95	0.019
**Glasgow coma scale score [*****n*** **(%)]**				
< 11	35 (17.5%)	12 (8.2%)	23 (43.4%)	< 0.001
≥11	165 (82.5%)	135 (91.8%)	30 (56.6%)	
**With intraventricular hemorrhage [*****n*** **(%)]**				
No	112 (56.0%)	95 (64.6%)	17 (32.1%)	< 0.001
Yes	88 (44.0%)	52 (35.4%)	36 (67.9%)	
**Hunt-Hess grade [*****n*** **(%)]**				
I–II	133 (66.5%)	115 (78.2%)	18 (34.0%)	< 0.001
III–V	67 (33.5%)	32 (21.8%)	35 (66.0%)	
**Fisher grade [*****n*** **(%)]**				
I–II	128 (64.0%)	113 (76.9%)	15 (28.3%)	< 0.001
III–IV	72 (36.0%)	34 (23.1%)	38 (71.7%)	
**Surgical mode [*****n*** **(%)]**				
Embolization	165 (82.5%)	131 (89.1%)	34 (64.2%)	< 0.001
Craniotomy	35 (17.5%)	16 (10.9%)	19 (35.8%)	
**Hypertension**				
No	93 (46.5%)	75 (51.0%)	18 (34.0%)	< 0.001
Yes	107 (53.5%)	72 (49.0%)	35 (66.0%)	
**Diabetes**				
No	174 (87.0%)	127(86.4%)	47 (88.7%)	0.672
Yes	26 (13.0%)	20(13.6%)	6 (11.3%)	
**Location of aneurysm**				
Anterior circulation aneurysms [*n* (%)]	187 (93.5%)	139 (94.6%)	48 (90.6%)	0.312
Posterior circulation aneurysms [*n* (%)]	13 (6.5%)	8 (5.4%)	5 (9.4%)	
**Coronary atherosclerotic heart disease [*****n*** **(%)]**				
No	193 (96.5%)	143 (97.3%)	50 (94.3%)	0.574
Yes	7 (3.5%)	4 (2.7%)	3 (5.7%)	
**Infections [*****n*** **(%)]**				
No	62 (31.0%)	56 (38.1%)	6 (11.3%)	< 0.001
Yes	138 (69.0%)	91 (61.9%)	47 (88.7%)	

Among all included aSAH patients, the neutrophil level on the first postoperative day was 9.28 ± 5.00 × 10^9^/L, the lymphocyte level was 0.90 ± 0.57 × 10^9^/L, the SIRI level was 7.18 ± 8.44 × 10^9^/L, and the NLR level was 10.72 ± 8.24. On the seventh postoperative day, the neutrophil level was 7.49 ± 4.45 × 10^9^/L, the lymphocyte level was 1.26 ± 0.71 × 10^9^/L, the SIRI level was 4.17 ± 4.23 × 10^9^/L, and the NLR level was 5.81 ± 4.50. The red blood cell count in cerebrospinal fluid on the first postoperative day was 129 ± 253 × 10^9^/L, and was 34.5 ± 77.3 × 10^9^/L on the seventh postoperative day. The white blood cell count in cerebrospinal fluid on the first postoperative day and seventh postoperative day were 0.146 ± 0.323 × 10^9^/L and 0.243 ± 0.493 × 10^9^/L, respectively (Table 2).

**Table 2:** Inflammatory markers and blood cell counts in cerebrospinal fluid in all included patients.

	**Total (*n* = 200)**	**Good outcome (*n* = 147)**	**Poor outcome (*n* = 53)**	***P-*value**
**Peripheral blood cell counts and inflammatory markers**				
Neutrophil on the 1st postoperative day ( × 10^9^/L)	9.28 ± 5.00	8.82 ± 4.49	11.34 ± 5.55	< 0.001
Lymphocyte on the 1st postoperative day ( × 10^9^/L)	0.90 ± 0.57	0.95 ± 0.60	0.82 ± 0.43	0.081
System inflammation response index on the 1st postoperative day ( × 10^9^/L)	7.18 ± 8.44	5.87 ± 6.56	10.21 ± 8.83	< 0.001
Systemic immune inflammation index on the 1st postoperative day ( × 10^9^/L)	1,891.87 ± 1,652.47	1,787.56 ± 1,564.96	2,385.61 ± 1,788.81	0.001
Neutrophil-lymphocyte ratio on the 1st postoperative day	10.72 ± 8.24	9.94 ± 7.57	14.05 ± 11.59	< 0.001
Platelet-lymphocyte ratio on the 1st postoperative day	200.04 ± 126.24	196.55 ± 116.43	212.20 ± 167.06	0.275
Neutrophil on the 7th postoperative day ( × 10^9^/L)	7.49 ± 4.45	6.95 ± 3.33	10.66 ± 4.59	< 0.001
Lymphocyte on the 7th postoperative day ( × 10^9^/L)	1.26 ± 0.71	1.30 ± 0.67	1.09 ± 0.68	0.017
System inflammation response index on the 7th postoperative day ( × 10^9^/L)	4.17 ± 4.23	3.64 ± 3.08	7.28 ± 8.78	< 0.001
Systemic immune inflammation index on the 7th postoperative day ( × 10^9^/L)	1,272.53 ± 1,136.51	1,212.70 ± 938.36	1,977.70 ± 1,762.96	< 0.001
Neutrophil-lymphocyte ratio on the 7th postoperative day	5.81 ± 4.50	6.95 ± 3.33	9.48 ± 9.30	< 0.001
Platelet-lymphocyte ratio on the 7th postoperative day	171.05 ± 102.84	167.46 ± 94.80	192.12 ± 138.35	0.416
**Blood cell counts in cerebrospinal fluid**				
White blood cell count in cerebrospinal fluid on the 1st postoperative day ( × 10^9^/L)	0.146 ± 0.323	0.119 ± 0.260	0.400 ± 0.607	< 0.001
Red blood cell count in cerebrospinal fluid on the 1st postoperative day ( × 10^9^/L)	129 ± 253	103 ± 200	311 ± 221	< 0.001
White blood cell count in cerebrospinal fluid on the 7th postoperative day ( × 10^9^/L)	0.243 ± 0.493	0.133 ± 0.348	0.451 ± 0.866	0.001
Red blood cell count in cerebrospinal fluid on the 7th postoperative day ( × 10^9^/L)	34.5 ± 77.3	26.0 ± 35.8	86.5 ± 202.8	< 0.001

According to the mRS score, there were 147 patients with good outcome and 53 patients with poor outcome. The average age in patients with poor outcome was 62 years old, older than patients with good outcome (59 years), *P* = 0.019. Among patients with poor outcome, there were 66.0% cases with hypertension, more than patients with good outcome (49.0%), *P* < 0.001. The proportions of cases with GCS score < 11 (43.4 vs. 8.2%, *P* < 0.001), with ventricular hemorrhage (67.9 vs. 35.4%, *P* < 0.001), with Hunt-Hess grade III–V (66.0 vs. 21.8%, *P* < 0.001), and with Fisher grade III–IV (71.7 vs. 23.1%, *P* < 0.001) in patients with poor outcome were all significantly higher than patients with good outcome. Among patients with poor outcome, there were 19 (35.8%) cases undergoing craniotomy surgeries, which was also significantly higher than patients with good outcome (10.9%), *P* < 0.001. Patients with poor outcome also had higher proportion of infections (88.7 vs. 61.9%, *P* < 0.001). The surgical duration in patients with poor outcome was 180 ± 95 min, significantly longer than patients with good outcome (160 ± 70 min), *P* = 0.019. There were no significant differences in the proportions of males (*P* = 0.080), cases with posterior circulation aneurysms (*P* = 0.312), diabetes (*P* = 0.672), and coronary atherosclerotic heart disease (*P* = 0.574) between patients with good outcome and poor outcome (Table 1).

On the first postoperative day, the neutrophil level in patients with poor outcome was 11.34 ± 5.55 × 10^9^/L, significantly higher than patients with good outcome (8.82 ± 4.49 × 10^9^/L), *P* < 0.001. The NLR level in patients with poor outcome was 14.05 ± 11.59, significantly higher than patients with good outcome (9.94 ± 7.57), *P* < 0.001. The SIRI level (10.21 ± 8.83 × 10^9^/L vs. 5.87 ± 6.56 × 10^9^/L, *P* < 0.001) and SII level (2,385.61 ± 1,788.81 × 10^9^/L vs. 1,787.56 ± 1,564.96 × 10^9^/L, *P* = 0.001) in patients with poor outcome were all significantly higher than patients with good outcome. The red blood cell count in cerebrospinal fluid among patients with poor outcome was 311 ± 221 × 10^9^/L, significantly higher than patients with good outcome (103 ± 200 × 10^9^/L), *P* < 0.001. The white blood cell count in cerebrospinal fluid among patients with poor outcome was 0.400 ± 0.607 × 10^9^/L, significantly higher than patients with good outcome (0.119 ± 0.260 × 10^9^/L), *P* < 0.001 (Table 2).

On the seventh postoperative day, the neutrophil level in patients with poor outcome was 10.66 ± 4.59 × 10^9^/L, significantly higher than patients with good outcome (6.95 ± 3.33 × 10^9^/L), *P* < 0.001. The lymphocyte level was 1.09 ± 0.68 × 10^9^/L, lower than patients with good outcome (1.30 ± 0.67 × 10^9^/L), *P* = 0.017. The NLR level in patients with poor outcome was 9.48 ± 9.30, significantly higher than patients with good outcome (5.51 ± 3.48), *P* < 0.001. The SIRI level (7.28 ± 8.78 × 10^9^/L vs. 3.64 ± 3.08 × 10^9^/L, *P* < 0.001) and SII level (1,977.70 ± 1,762.96 × 10^9^/L vs. 1,212.70 ± 938.36 × 10^9^/L, *P* < 0.001) in patients with poor outcome were all significantly higher than patients with good outcome. The red blood cell count in cerebrospinal fluid among patients with poor outcome was 86.5 ± 202.8 × 10^9^/L, significantly higher than patients with good outcome (26.0 ± 35.8 × 10^9^/L), *P* < 0.001. The white blood cell count in cerebrospinal fluid among patients with poor outcome was 0.451 ± 0.866 × 10^9^/L, significantly higher than patients with good outcome (0.133 ± 0.348 × 10^9^/L), *P* = 0.001 (Table 2).

We used the receiver operating characteristic curve for the cut-off value. The cut-off value of surgery duration was 169 min (AUC = 0.608, 95% CI: 0.516–0.701, Youden index = 0.282, *P* = 0.019). The cut-off value of red blood cell count in cerebrospinal fluid on the first postoperative day was 177 × 10^9^/L (AUC = 0.733, 95% CI: 0.629–0.837, Youden index = 0.493, *P* < 0.001), with sensitivity of 82.6% and specificity of 66.7%. The cut-off value of NLR level on the seventh postoperative day was 8.16 (AUC = 0.756, 95% CI: 0.670–0.843, Youden index = 0.481, *P* < 0.001), with sensitivity of 63.6% and specificity of 84.5%. The cut-off value of red blood cell count in cerebrospinal fluid was 54 × 10^9^/L (AUC = 0.779, 95% CI: 0.671–0.887, Youden index = 0.527, *P* < 0.001), with sensitivity of 66.7% and specificity of 86.0% (Table 3).

**Table 3:** The results of area under the curve and cut-off values by the receiver operating characteristic curves.

	**Cut-off value**	**Area under the curve (95% CI)**	**Youden index**	***P-*value**	**Sensitivity**	**Specificity**
**On the 1st postoperative day**						
Neutrophil ( × 10^9^/L)	10.40	0.705 (0.622–0.788)	0.366	< 0.001	67.9%	68.7%
System inflammation response index ( × 10^9^/L)	5.9	0.678 (0.594–0.762)	0.315	< 0.001	81.10%	50.3%
Systemic immune inflammation index ( × 10^9^/L)	2,054.51	0.659 (0.576–0.743)	0.279	0.001	66.00%	61.9%
Neutrophil-lymphocyte ratio	11.24	0.678 (0.597–0.759)	0.305	< 0.001	67.90%	62.6%
RBCs in cerebrospinal fluid ( × 10^9^/L)	177	0.733 (0.629–0.837)	0.493	< 0.001	82.60%	66.7%
WBCs in cerebrospinal fluid ( × 10^9^/L)	0.180	0.761 (0.664–0.857)	0.511	< 0.001	87.00%	64.2%
**On the 7th postoperative day**						
Neutrophil ( × 10^9^/L)	9.96	0.755 (0.670–0.841)	0.490	< 0.001	65.9%	83.1%
lymphocyte ( × 10^9^/L)	1.11	0.619 (0.524–0.715)	0.207	0.017	66.2%	54.5%
System inflammation response index ( × 10^9^/L)	5.89	0.779 (0.698–0.860)	0.507	< 0.001	70.50%	80.3%
Systemic immune inflammation index ( × 10^9^/L)	1,894.37	0.688 (0.593–0.783)	0.347	< 0.001	52.30%	82.4%
Neutrophil-lymphocyte ratio	8.16	0.756 (0.670–0.843)	0.481	< 0.001	63.60%	84.5%
RBCs in cerebrospinal fluid ( × 10^9^/L)	54	0.779 (0.671–0.887)	0.527	< 0.001	66.70%	86.0%
WBCs in cerebrospinal fluid ( × 10^9^/L)	0.192	0.715 (0.599–0.831)	0.347	0.001	76.70%	58.0%

**Figure 2:**
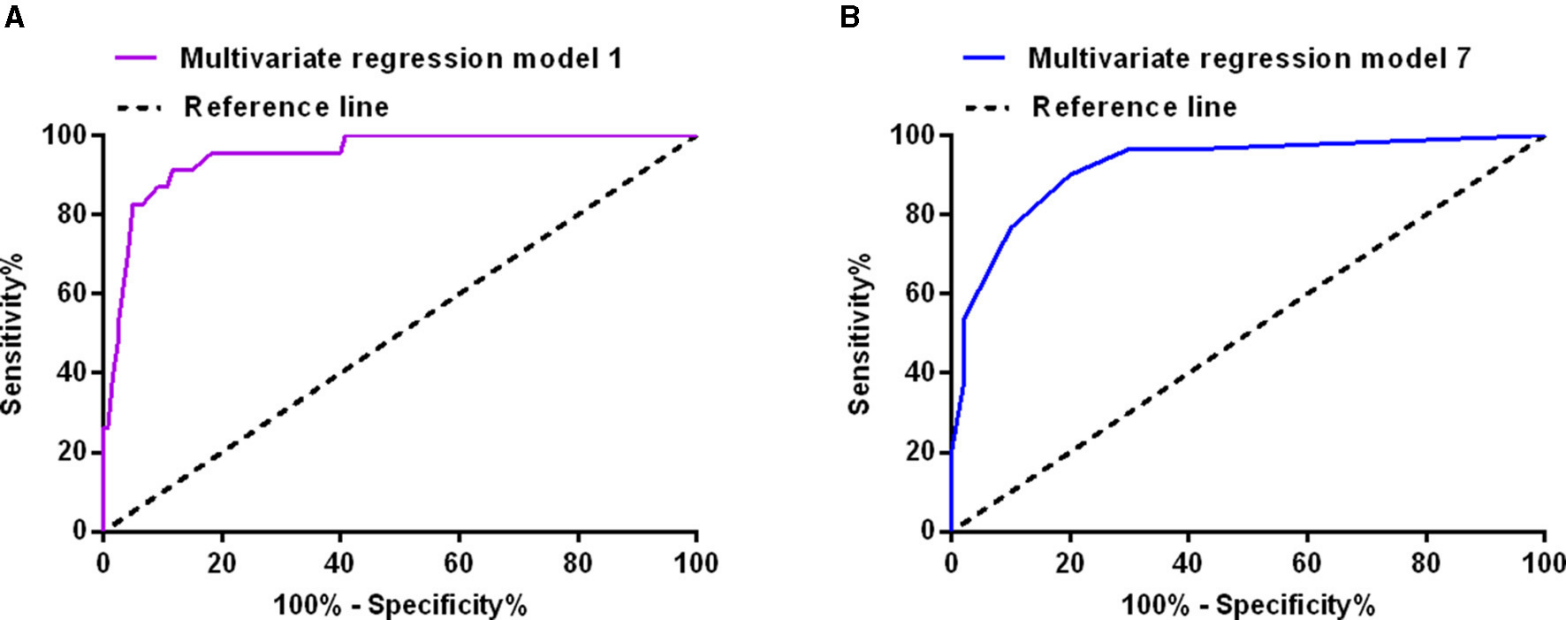
The receiver operating characteristic curves of the multivariate logistic regression model including inflammation markers and blood cell count in cerebrospinal fluid on the first postoperative day **(A)**, and on the seventh postoperative day **(B)**.

The multivariate logistic regression model including inflammatory markers and blood cell counts in cerebrospinal fluid on the 1st postoperative day confirmed that red blood cell count in cerebrospinal fluid (≥177 × 10^9^/L; OR: 7.227, 95% CI: 1.160–45.050, *P* = 0.034) was possibly associated with poor outcome of aSAH patients. Surgical duration (≥169 min), Fisher grade (III–IV), hypertension, and infections were also possibly associated with poor outcome of aSAH patients (Table 4). The sensitivity and specificity of the model were 91.3 and 88.3%, respectively (AUC = 0.951, 95% CI: 0.909–0.992, Youden index = 0.796, *P* < 0.001; Figure 2A). The model including inflammatory markers and blood cell counts in cerebrospinal fluid on the 7th postoperative day confirmed that red blood cell count in cerebrospinal fluid (≥54 × 10^9^/L; OR: 39.787, 95% CI: 6.799–232.836, *P* < 0.001) and neutrophil-lymphocyte ratio (≥8.16; OR: 6.362, 95% CI: 1.424–28.428, *P* = 0.015) were all possibly associated with poor outcome of aSAH patients (Table 4). The sensitivity and specificity of this model were 90.0 and 80.0%, respectively (AUC = 0.921, 95% CI: 0.857–0.984, Youden index = 0.700, *P* < 0.001; Figure 2B).

**Table 4:** Factors associated with poor outcome in aneurysmal subarachnoid hemorrhage patients by multivariate logistic regression models.

**Models**	**Variable**	**B**	**S.E**.	**Wald**	***P-*value**	**Adjusted odds ratio (95% CI)**
**Multivariate logistic regression model including inflammatory markers and blood cell counts in cerebrospinal fluid on the 1st postoperative day**						
1	Surgical duration (≥169 min)	2.097	0.810	6.703	0.010	8.144 (1.665–39.845)
	Fisher grade (III–IV)	2.991	0.838	12.749	< 0.001	19.904 (3.854–102.794)
	Hypertension	2.437	0.912	7.150	0.007	11.443 (1.917–68.302)
	Infections	2.719	1.057	6.620	0.010	15.160 (1.911–120.253)
	White blood cell count in cerebrospinal fluid (≥0.180 × 10^9^/L)	1.910	0.976	3.826	0.050	6.753(0.996–45.776)
	Red blood cell count in cerebrospinal fluid (≥177 × 10^9^/L)	1.978	0.934	4.488	0.034	7.227 (1.160–45.050)
**Multivariate logistic regression model including inflammatory markers and blood cell counts in cerebrospinal fluid on the 7th postoperative day**						
2	Neutrophil (≥9.96 × 10^9^/L)	2.496	0.842	8.786	0.003	12.135 (2.329–63.218)
	Neutrophil-lymphocyte ratio (≥8.16)	1.850	0.764	5.869	0.015	6.362 (1.424–28.428)
	Red blood cell count (≥54 × 10^9^/L)	3.684	0.901	16.698	< 0.001	39.787(6.799–232.836)

**Figure 3:**
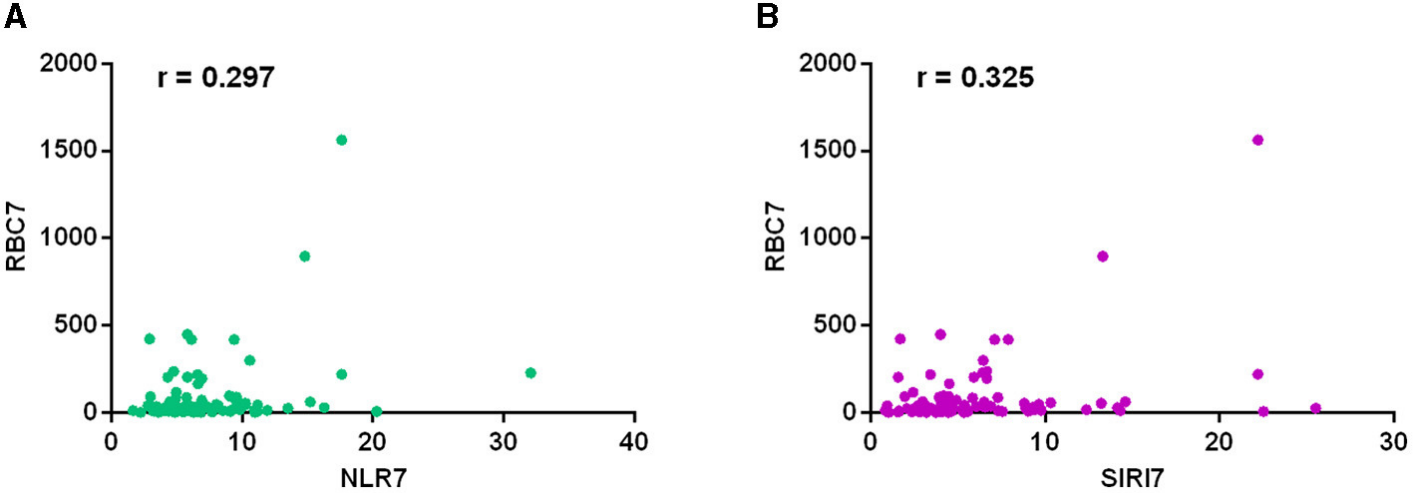
The correlation analysis between inflammatory marker and cell count in cerebrospinal fluid on the seventh postoperative day. The results showed that the NLR (*r* = 0.297, *P* = 0.007) **(A)** and SIRI (*r* = 0.325, *P* = 0.003) **(B)** levels were all correlated with the count of red blood cells in cerebrospinal fluid.

We conducted a correlation analysis between inflammatory marker and blood cell count in cerebrospinal fluid respectively on the first postoperative day and seventh postoperative day. The results on the 7th postoperative day showed that the NLR (*r* = 0.297, *P* = 0.007) and SIRI (*r* = 0.325, *P* = 0.003) levels were all correlated with the count of red blood cells in cerebrospinal fluid (Figure 3). The results on the 1st postoperative day showed that NLR level (*r* = 0.173, *P* = 0.039) was correlated with the count of white blood cells in cerebrospinal fluid. The SIRI level (*r* = 0.337, *P* = 0.002) was also correlated with the count of white blood cells in cerebrospinal fluid on the 7th postoperative day. No correlations were found between other inflammatory markers and cell count in cerebrospinal fluid (Table 5).

**Table 5:** The results of correlation analysis between inflammatory marker and blood cell count in cerebrospinal fluid.

**On the 7th postoperative day**							
		**N**	**L**	**NLR**	**SIRI**	**SII**	**PLR**
Red blood cell count in CSF	*r*	0.091	−0.199	0.297	0.325	0.193	0.141
	*P*-value	0.422	0.077	0.007	0.003	0.087	0.211
White blood cell count in CSF	*r*	0.116	−0.101	0.215	0.337	0.114	0.049
	*P*-value	0.306	0.373	0.056	0.002	0.313	0.664
**On the 1st postoperative day**							
		**N**	**L**	**NLR**	**SIRI**	**SII**	**PLR**
Red blood cell count in CSF	*r*	0.088	−0.063	0.109	0.064	0.001	−0.048
White blood cell count in CSF	*P*-value	0.295	0.454	0.196	0.448	0.991	0.569
Red blood cell count in CSF	*r*	0.139	−0.077	0.173	0.11	0.053	−0.027
White blood cell count in CSF	*P*-value	0.099	0.362	0.039	0.191	0.526	0.752

CSF, cerebrospinal fluid; N, neutrophil; L, lymphocyte; NLR, neutrophil-lymphocyte ratio; SIRI, system inflammation response index; SII, systemic immune inflammation index.

## Discussion

Aneurysmal subarachnoid hemorrhage is a highly complex and fatal disease. Although significant progression has been made in the treatment of aSAH in recent years, the outcome of some patients is still poor. In recent years, many studies found that neuroinflammation played an important role in the progression of subarachnoid hemorrhage (7, 8). Therefore, many studies have begun to explore the associations between inflammatory markers and clinical outcome of patients.

NLR is the ratio of neutrophil to lymphocyte, which has become a research focus due to its ability to reflect the changes of neutrophil and lymphocyte. The NLR level was shown to be associated with the 90-d outcome in patients with cerebral hemorrhage, and also associated with GCS score and amount of hemorrhage (9). Higher NLR level was also a risk factor for 90-d mortality in patients with cerebral hemorrhage (4). In patients with aSAH, cases with poor outcome had significantly higher NLR level at admission, which was an important factor associated with poor outcome in these patients (3). Different from this study, we found a significant increase of NLR level on the first and seventh postoperative days. Moreover, the multivariate logistic regression model also showed that NLR ≥ 8.16 on the seventh postoperative day was associated with poor outcome of aSAH patients.

In our study, we also explored the associations between other inflammatory markers and the 90-d outcome of patients. PLR is the ratio of platelet to lymphocyte. It was not only associated with the outcome of many tumor patients, but also associated with the response of tumor patients to anti-tumor therapy (10, 11). Among patients with acute ischemic stroke, higher PLR was possibly associated with poor outcome, reperfusion insufficiency, and infarct size (12). The SII marker integrates platelet, neutrophil, and lymphocyte. It was possibly associated with the severity of some diseases and the outcome of cancer patients (13–15). A meta-analysis including 19 studies and 18,609 stroke patients showed that higher SII was possibly associated with poor outcome of stroke patients (16). SIRS integrates neutrophil, monocyte, and lymphocyte. Some studies also supported the association between SIRI level and the survival of cancer patients (17, 18). In our study, all multivariate logistic regression models did not confirm the associations of PLR, SII, SIRI, and lymphocyte with poor outcome of aSAH patients.

As many studies have shown, hemoglobin released by red blood cells possibly induced cerebral vasospasm, further affected the outcome of cerebral hemorrhage patients. Nozaki et al. detected the spasmodic activity of various blood components in dogs and showed that red blood cells were possibly required for late and prolonged arterial spasms after subarachnoid hemorrhage (19). Moreover, patients with delayed cerebral ischemia showed a significant increase of red blood cells in cerebrospinal fluid (20). Our study analyzed the associations of red blood cells and white blood cells in cerebrospinal fluid on the first and seventh postoperative days with clinical outcome. The multivariate model including inflammatory markers and blood cell count in cerebrospinal fluid on the first and seventh postoperative days all showed that higher red blood cell count in cerebrospinal fluid was possibly associated with poor outcome of aSAH patients.

We also conducted the correlation analysis between red blood cell count in cerebrospinal fluid and inflammatory markers (including NLR, SIRI, PLR, and SII) on the first and seventh postoperative days. The results showed a mild correlation of red blood cell count in cerebrospinal fluid with NLR and SIRI levels on the seventh postoperative day. We speculated that red blood cell count in cerebrospinal fluid could reflect the inflammatory response status in aSAH patients. Systemic inflammation after hemorrhagic stroke possibly played an important role in inducing intracranial and extracranial tissue damage (21–23). After occurrence of aneurysmal subarachnoid hemorrhage, the blood accumulated in the subarachnoid space, and then red blood cells underwent hemolysis and degradation. Oxidized hemoglobin induced systemic inflammation through a series of cytokines and multiple signaling pathways, leading to the contraction of vascular smooth muscle, further cerebral vasospasm and ultimately delayed brain injury (24–29).

In our study, patient with poor outcome had more surgical craniotomy, longer surgical duration and higher systemic inflammatory response, we speculated that the surgical approach was possibly associated with longer operation time, more intracerebral bleeding, or more infections. We also speculated that a less invasive approach might correlate with few complications and a weak pro-inflammatory response. Other important message of our study was the beneficial effects of the less invasive approach, compared with craniotomy.

Our study had several limitations. Firstly, due to the limitation of retrospective nature, cerebrospinal fluid sample on the seventh postoperative day was relatively small, which could lead to some bias. We will expand the sample size in the future study. Secondly, we only collected cerebrospinal fluid data on the first and seventh postoperative day, did not study the dynamic changes of inflammatory markers. We will explore the associations of dynamic inflammatory marker and blood cell count in cerebrospinal fluid with the outcome of aSAH patients. Due to the limitation of laboratory condition in the hospital, we were unable to detect hemoglobin levels in cerebrospinal fluid, which was also one limitation. An important limitation was the lack of any cytokine data including IL-6, IL-10, and TNF alfa in cerebrospinal fluid, which would have added insights to the study.

Higher neutrophil-lymphocyte ratio and higher red blood cell count in cerebrospinal fluid were all possibly associated with poor outcome of patients with aneurysmal subarachnoid hemorrhage. It is very important to conduct a study on reducing blood stimulation to the subarachnoid space and inflammatory response to improve clinical outcome of aneurysmal subarachnoid hemorrhage patients. We also need a larger sample study.

## Data availability statement

The original contributions presented in the study are included in the article/supplementary material, further inquiries can be directed to the corresponding author.

## Ethics statement

The studies involving humans were approved by the First Affiliated Hospital of Yangtze University. The studies were conducted in accordance with the local legislation and institutional requirements. Written informed consent for participation was not required from the participants or the participants' legal guardians/next of kin in accordance with the national legislation and institutional requirements. Written informed consent was obtained from the individual(s) for the publication of any potentially identifiable images or data included in this article.

## Author contributions

JM: Conceptualization, Supervision, Writing – original draft. YZ: Data curation, Software, Writing – review & editing. CL: Data curation, Writing – review & editing. HH: Data curation, Writing – review & editing.
